# Bevacizumab in first-line treatment of elderly patients with metastatic colorectal cancer: German community-based observational cohort study results

**DOI:** 10.1186/1471-2407-14-761

**Published:** 2014-10-13

**Authors:** Ralf Hofheinz, Volker Petersen, Manfred Kindler, Mathias Schulze, Joerg Seraphin, Heinz-Gert Hoeffkes, Anette-R Valdix, Jan Schroeder, Julia Herrenberger, Alexander Stein, Axel Hinke, Dirk Arnold

**Affiliations:** Interdisziplinäres Tumorzentrum Mannheim, Universitätsmedizin Mannheim, Theodor-Kutzer-Ufer 1-3, 68167 Mannheim, Germany; Private Practice, Iglauerstrasse 2, 89518 Heidenheim, Germany; Onkologische Schwerpunktpraxis, Landsberger Allee 277, 13055 Berlin, Germany; Ambulante Onkologie Zittau, Görlitzerstrasse 10a, 02763 Zittau, Germany; Haematologisch-Onkologische Schwerpunktpraxis Northeim, Sturmbäume 3, 37154 Northeim, Germany; Medizinisches Versorgungszentrum Fulda, Pacelliallee 4, 36045 Fulda, Germany; Onkologische Schwerpunktpraxis Schwerin, Kielerstrasse 31a, 19057 Schwerin, Germany; Schwerpunktpraxis für Hämatologie und Onkologie, Kettwigerstrasse 62, 45486 Mühlheim an der Ruhr, Germany; Onkologische Schwerpunktpraxis, Clayallee 225a, 14195 Berlin, Germany; Department of Oncology, Hematology, BMT with section Pneumology, University Medical Center Hamburg-Eppendorf, Martinistrasse 52, 20246 Hamburg, Germany; WiSP Research Institute, Karl-Benz-Strasse 1, 40764 Langenfeld, Germany; Department of Medical Oncology, Klinik fuer Tumorbiologie, Breisacher Straße 117, 79106 Freiburg, Germany

**Keywords:** Bevacizumab, Metastatic colorectal cancer, Elderly, First-line, Observational cohort, Germany

## Abstract

**Background:**

To evaluate the efficacy of first-line bevacizumab-based chemotherapy for untreated metastatic colorectal cancer (mCRC) based on age.

**Methods:**

Eligibility criteria focused on M1 disease without prior palliative chemotherapy. Choice of chemotherapy regimen was at the physician’s discretion. Predefined efficacy endpoints were response rate, progression-free and overall survival (PFS, OS). Patients were analysed by age (<70 vs. ≥70 years, <75 vs. ≥75 years).

**Results:**

Of 1777 patients, 27% and 12% were ≥70 and ≥75 years, respectively. PFS was shorter in elderly patients (<70 vs. ≥70 years: 10.5 vs. 9.5 months, p = 0.074; <75 vs. ≥75 years: 10.5 vs. 8.9 months, p = 0.00019), as was OS (<70 vs. ≥70 years: 25.8 vs. 22.7 months, p < 0.0008; <75 vs. ≥75 years: 25.8 vs. 20.8 months; p < 0.0001). In the groups <70 and <75 years, PFS was longer in those receiving oxaliplatin-/irinotecan-containing regimens vs. those receiving 5-FU/capecitabine (<70 years: 10.6 vs. 9.0 months; p = 0.0065; <75 years: 10.6 vs. 9.2 months; p = 0.028); no difference in PFS was observed between oxaliplatin-/irinotecan-containing regimens vs. 5-FU/capecitabine regimens in both elderly age-group comparisons (≥70 years: 9.7 vs. 9.2 months; ≥75 years: 8.3 and 9.0 months).

**Conclusion:**

First-line bevacizumab-based chemotherapies were effective in German mCRC patients ≥75 years of age, but PFS and OS were significantly shorter in this age group vs. younger patients.

## Background

The incidence and prevalence of cancer are rising among older populations in developed countries [1] with more than 60% of all cancers being diagnosed in people >65 years of age [2, 3]. Focusing specifically on colorectal cancer, almost 75% of patients with the disease are >65 years of age and the median age at diagnosis is 70 years [4]. Despite this, older patients are typically under-represented in clinical trials, with <10% of patients enrolled in colorectal cancer clinical trials being >70 years of age [5].

In randomised trials involving patients with metastatic colorectal cancer (mCRC), the addition of the humanised monoclonal antibody bevacizumab to first- and second-line therapies has resulted in significantly improved progression-free survival (PFS) compared with chemotherapy alone [6–8]. Recently, the AVEX trial reported a clinically significant benefit of adding bevacizumab to low doses of capecitabine (2000 mg/m^2^/day) in patients aged ≥70 years not deemed suitable for treatment with chemotherapy doublets. In this study, patients with a median age of 76–77 years derived a significant 4-month benefit in PFS (hazard ratio: 0.53, 95% CI: 0.41–0.69; p < 0.001) and a clinically, but not statistically, significant overall survival (OS) benefit of 3.9 months (hazard ratio: 0.79, (95% CI: 0.57–1.09; p = 0.182) with bevacizumab + capecitabine over capecitabine alone [9].

In the randomised, open-label FOCUS2 trial, factorial comparison of 459 elderly and frail patients with advanced colorectal cancer found the addition of oxaliplatin versus no addition provided some improvement in PFS, but the finding was not significant (median 5.8 months [interquartile range: 3.3–7.5] vs 4.5 months [2.8–6.4]; hazard ratio 0.84, 95% CI: 0.69–1.01, p = 0 · 07), while replacement of fluorouracil with capecitabine did not improve global quality of life scores [10]. A pooled analysis of elderly mCRC patients from randomised clinical studies showed that the addition of bevacizumab to chemotherapy provided similar PFS and OS benefits in medically fit older patients as in younger patients [11]. Similarly, in the BRiTE prospective observational cohort study, which included 363 patients ≥65 years of age, elderly patients receiving bevacizumab had similar PFS as younger patients, although as expected OS diminished with increased age [12]. However, despite these findings there is still a relative paucity of data on the use of bevacizumab in daily clinical practice in patients >70 years and particularly for those who are >75 years of age.

Following the approval of bevacizumab in Germany in 2005 for the treatment of unresectable advanced or refractory CRC, an observational cohort study was initiated to assess the efficacy and safety of bevacizumab as part of first-line chemotherapy for mCRC in German patients. Analyses were also performed to investigate the efficacy and safety of treatment with bevacizumab plus chemotherapy in elderly patients (either ≥70 or ≥75 years) with mCRC compared with younger patients (<70 or <75 years, respectively).

## Methods

### Observational cohort design and patients

This was an observational cohort study of patients with mCRC who had received no prior chemotherapy for metastatic disease. To facilitate enrolment of a typical mCRC population, eligibility criteria were minimised. All patients scheduled to undergo first-line treatment with bevacizumab were included. The choice of chemotherapy regimen was at the physicians’ discretion, but was influenced by current registration status (i.e. 5-FU or capecitabine alone or in combination with oxaliplatin or irinotecan). The target was to recruit 1600 patients. Detailed information on baseline data, antineoplastic treatment, tumour development and safety were collected up to termination of bevacizumab therapy, or for a period of 12 months, in most cases. Thereafter, long-term assessment data on key parameters were retrieved repeatedly by additional fax forms for up to 6 years after initiation of treatment.

This was an observational study in which physicians’ choices were guided by drug registration status and treatment guidelines (rather than the trial protocol). As the study was started prior to 2007, it was in agreement with the German FSA Codex [13] and the AMG Amendment 12, there was no need/requirement for ethics committee approval or written informed consent. For non-interventional studies started in 2007 or later, the FSA Codex asks for submission to the ethics committee and to the regulators. Furthermore, in the European Union, clinical research has to be performed according to the Directive 2001/20/EC of the European Parliament and of the Council on the approximation of the laws, regulations and administrative provisions of the Member States relating to the implementation of good clinical practice in the conduct of clinical trials on medicinal products for human use dating from April 2001. This regulation differentiates between the requirements for “interventional” and “non-interventional” studies. This observational study clearly fulfills the criteria for “non-interventional” as defined in Article 2, c.

### Treatment

Patients received bevacizumab 5–10 mg/kg every 2 weeks or 7.5–15 mg/kg every 3 weeks in combination with chemotherapy; patients were also allowed to receive bevacizumab monotherapy.

### Endpoints

Predefined efficacy endpoints were investigator-assessed response rate (as best response, unconfirmed), PFS (time from start of first-line therapy to investigator-assessed progression or death, whichever occurred first), and OS (time from start of first bevacizumab administration to death). Adverse events potentially related to antibody treatment were recorded (by use of open questions) and assessed, especially those of interest for bevacizumab, such as hypertension, proteinuria, gastrointestinal perforation, haemorrhage, and arterial/venous thromboembolic events. An adverse drug reaction was defined as an event for which a causal relationship with bevacizumab could not be ruled out or was unknown. A serious adverse drug reaction was defined as any event that resulted in death, was incapacitating, or required inpatient hospitalisation/prolongation of existing hospitalisation for treatment.

### Data analysis

Database lock occurred in November 2011. Exploratory post-hoc subgroup analyses were performed to evaluate the efficacy of treatment in patients ≥70 and those <70 years of age, and those ≥75 and <75 years of age. Analyses were based on the population of patients who had received at least one dose of bevacizumab. PFS and OS were recorded based on investigators’ evaluation (with the assessment schedule at investigators’ discretion) and analysed using Kaplan–Meier methodology, with median survival times and 95% confidence intervals (CIs). The curves were compared using two-sided log-rank tests. The rate of adverse events (all types and those of special interest for bevacizumab) were presented descriptively and summarised by study treatment.

## Results

### Patients

Between January 2005 and June 2009, 1777 eligible patients were enrolled at 261 sites in Germany; of these, 480 (27%) and 206 patients (12%) were ≥70 and ≥75 years of age, respectively. Baseline characteristics according to patient age are shown in Table 1. Elderly patients in either age group did not differ greatly from younger patients in terms of: time from initial diagnosis or time to first relapse; initial pT, pN and M stage; site of metastasis; grading; carcinoembryonic antigen (CEA) level; white blood cell count; and blood pressure. Fewer patients aged ≥75 years vs. <75 years had received prior radiotherapy (12% vs. 18%), although this difference was slightly less in patients aged ≥70 years vs. <70 years (14% vs. 18%). There were no differences between groups with respect to previous (neo)adjuvant chemotherapy. Fewer elderly patients in both age groups had >1 organ involved and elderly patients, again in both age groups, tended to have poorer Eastern Co-operative Oncology Group (ECOG) performance status (Table 1).

**Table 1:** **Patients’ baseline characteristics according to age**

Characteristic	Age <70 years (n = 1297)	Age ≥70 years (n = 480)	Age <75 years (n = 1571)	Age ≥75 years (n = 206)
*Gender, n (%)*				
Male	811 (63)	297 (62)	985 (63)	123 (60)
Female	486 (37)	183 (38)	586 (37)	83 (40)
*Median age, years (range)*	61 (19–69)	73 (70–100)	63 (19–74)	77 (75–100)
*ECOG performance status, n (%)*				
0	503 (39)	153 (32)	598 (38)	58 (28)
1	647 (50)	251 (52)	785 (50)	113 (55)
2	119 (9)	56 (12)	147 (9)	28 (14)
3	4 (<1)	11 (2)	11 (1)	4 (2)
4	0	1 (<1)	1 (<1)	0
Missing	24 (2)	8 (2)	29 (2)	3 (1)
*Metastatic site, n (%)* ^*a*^				
Liver	935 (72)	332 (69)	1130 (72)	137 (67)
Lung	357 (28)	133 (28)	434 (28)	56 (27)
Bone	47 (4)	10 (2)	53 (3)	4 (2)
Other	350 (27)	107 (22)	408 (26)	49 (24)
*No. of metastatic sites, n (%)*				
1	824 (64)	321 (67)	1007 (64)	138 (67)
>1	405 (31)	124 (26)	476 (30)	53 (26)
Missing	68 (5)	35 (7)	88 (6)	15 (7)
*Local recurrence, n (%)*	585 (51)	220 (51)	711 (51)	94 (50)
*Median CEA, ng/mL (range)*	20.9 (0–14 671)	20.7 (0.1–25305)	20.4 (0–25 305)	25.6 (0.1–6848)
*Surgical removal of primary tumour, n (%)*	1210 (93)	442 (92)	1464 (93)	188 (91)
*Prior radiotherapy, %*	18	14	18	12
*Prior chemotherapy, %*				
*Adjuvant*	67	68	67	66
*Neoadjuvant*	11	11	10	12
*Systolic hypertension, %*				
*Mild*	26	28	27	25
*Moderate*	6	8	6	10
*Severe*	1	1	1	1
*Diaystolic hypertension, %*				
*Mild*	17	14	17	12
*Moderate*	2	3	2	3
*Severe*	1	1	1	0

CEA, carcinoembryonic antigen; ECOG, Eastern Cooperative Oncology Group. ^a^Patients could have >1 metastatic site.

### Treatment

**Figure 1:**
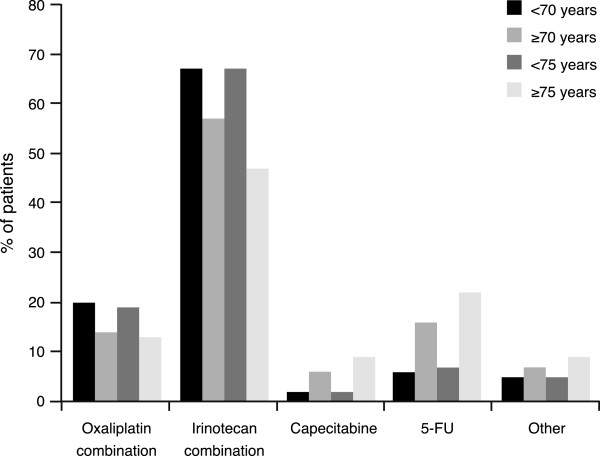
**Bevacizumab-based therapy administered in cycle 1 according to patient age.**

The median duration of treatment was 7 months in all four groups; patients <70, ≥70 and <75 years of age received a median of 8 treatment cycles while those aged ≥75 years received a median of 7.5 cycles. The differences between the younger and older age groups in the treatment chemotherapy backbone received, irrespective of whether the analysis was performed at <70 vs. ≥70 or <75 vs. ≥75 years, were: fewer older patients received doublet combinations at the start of treatment while more older patients received 5-FU or capecitabine monotherapy (Figure 1). Patients’ baseline characteristics by chemotherapy regimen and age are shown in Table 2.

**Table 2:** **Patients’ baseline characteristics according to chemotherapy regimen and age**

	Age <70 years		Age ≥70 years		Age <75 years		Age ≥75 years	
Characteristic	Monotherapy (n = 105)	Doublet (n = 1165)	Monotherapy (n = 104)	Doublet (n = 367)	Monotherapy (n = 146)	Doublet (n = 1396)	Monotherapy (n = 63)	Doublet (n = 136)
*Gender, n (%)*								
Male	60 (57)	739 (63)	60 (58)	234 (64)	84 (58)	888 (64)	36 (57)	85 (62)
Female	45 (43)	426 (37)	44 (42)	133 (36)	62 (42)	508 (36)	27 (43)	51 (38)
*Median age, years (range)*	62 (23–69)	61 (19–69)	76 (70–100)	73 (70–85)	65 (23–74)	63 (19–74)	78 (75–100)	77 (75–85)
*ECOG performance status, n (%)*	(n = 103)	(n = 1144)	(n = 101)	(n = 362)	(n = 142)	(n = 1372)	(n = 62)	(n = 134)
0	52 (50)	435 (38)	38 (38)	112 (31)	67 (47)	514 (37)	23 (37)	33 (25)
1	38 (37)	599 (52)	48 (48)	199 (55)	56 (39)	719 (52)	30 (48)	79 (59)
2	11 (11)	108 (9)	13 (13)	41 (11)	17 (12)	129 (9)	7 (11)	20 (15)
3	2 (2)	2 (<1)	2 (2)	9 (2)	2 (1)	9 (1)	2 (3)	2 (1)
4	0	0	0	1 (<1)	0	1 (0)	0	0
*Metastatic site, n (%)* ^*a*^								
Liver	69 (66)	849 (73)	73 (70)	253 (69)	98 (67)	1014 (73)	44 (70)	88 (64)
Lung	29 (28)	323 (28)	31 (30)	100 (27)	42 (29)	385 (28)	18 (29)	38 (28)
Bone	1 (1)	45 (4)	2 (2)	8 (2)	2 (1)	50 (4)	1 (2)	3 (2)
Other	32 (30)	311 (27)	22 (21)	82 (22)	41 (28)	361 (25)	14 (22)	32 (23)
*No. of metastatic sites, n (%)*	(n = 100)	(n = 1105)	(n = 102)	(n = 335)	(n = 140)	(n = 1317)	(n = 62)	(n = 123)
1	73 (73)	732 (66)	78 (76)	238 (71)	104 (74)	883 (67)	47 (76)	87 (71)
>1	27 (27)	373 (34)	24 (24)	97 (29)	36 (26)	434 (33)	15 (24)	36 (29)
*Local recurrence, %*	44 (45)	526 (51)	40 (42)	176 (53)	58 (42)	638 (51)	26 (46)	64 (51)
*Median CEA, ng/mL (range)*	(n = 92)	(n = 926)	(n = 91)	(n = 302)	(n = 126)	(n = 1113)	(n = 57)	(n = 115)
	19.8 (0.4–3312.7)	21.2 (0–14671)	13.7 (0.1–1175)	21.9 (0.7–25305)	17.2 (0.4–3312.7)	20.9 (0–25305)	22.7 (0.1–1175)	25.6 (0.7–6848)
*Prior chemotherapy, n (%)*	(n = 53)	(n = 670)	(n = 47)	(n = 190)	(n = 73)	(n = 786)	(n = 27)	(n = 74)
*Adjuvant*	35 (66)	453 (68)	33 (70)	127 (67)	47 (64)	534 (68)	21 (78)	46 (62)
*Neoadjuvant*	9 (17)	67 (10)	2 (4)	24 (13)	10 (14)	79 (10)	1 (4)	12 (16)

CEA, carcinoembryonic antigen; ECOG, Eastern Cooperative Oncology Group. ^a^Patients could have >1 metastatic site.

### Efficacy

In the overall/intent-to-treat (ITT) patient population, the best objective response rate following bevacizumab-based treatment was 60% (95% CI 58–63%; complete response 10%, partial response 51%). The investigator-assessed overall objective response rate (including all treatment regimens) in patients aged <70 years was significantly greater than that in those aged ≥70 years (62% vs. 55%; p = 0.0046 Fisher’s exact test; Table 3); similar findings were observed in patients aged <75 years vs. those aged ≥75 years (61% vs. 51%; p = 0.0041 Fisher’s exact test; Table 3).

**Figure 2:**
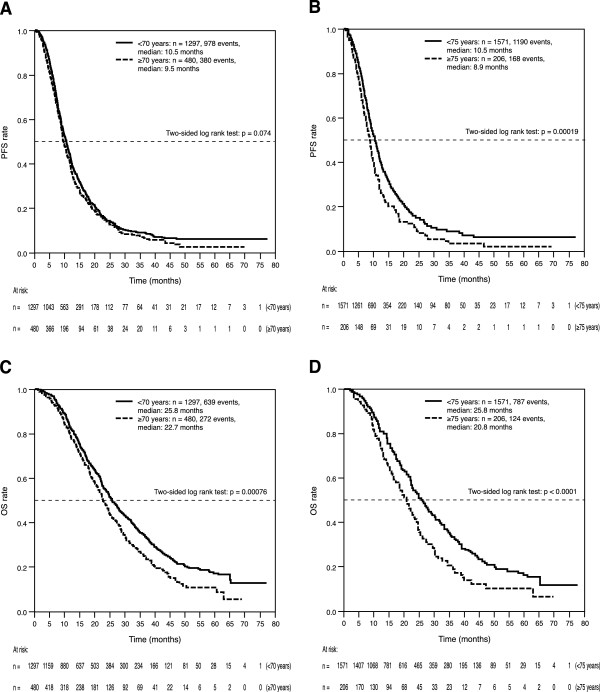
**Progression-free (PFS) and overall survival (OS) according to age. (A)** PFS according to Age (<70 and ≥70 years); **(B)** PFS according to Age (<75 and ≥75 years); **(C)** OS according to Age (<70 and ≥70 years); **(D)** OS according to Age (<75 and ≥75 years). OS: overall survival, PFS: progression-free survival.

Median PFS in the ITT population was 10.2 months, based on 1390 observed events. Median PFS in patients aged <70 years was longer than that in those aged ≥70 years (10.5 vs. 9.5 months; hazard ratio [HR]: 1.11, 95% CI: 0.99–1.25; 2-sided log-rank test p = 0.074; Figure 2A) and this reached statistical significance in patients <75 vs. ≥75 years of age (10.5 vs. 8.9 months; HR: 1.36, 95% CI: 1.16–1.60; 2-sided log-rank test p = 0.00019; Figure 2B). Corresponding values for median OS for patients aged <70 vs. ≥70 years and <75 vs. ≥75 years were 25.8 vs. 22.7 months (HR: 1.28, 95% CI: 1.11–1.47; 2-sided log-rank test p <0.0008; Figure 2C) and 25.8 vs. 20.8 months, respectively (HR: 1.48, 95% CI: 1.23–1.80; 2-sided log-rank test p <0.0001; Figure 2D).

**Figure 3:**
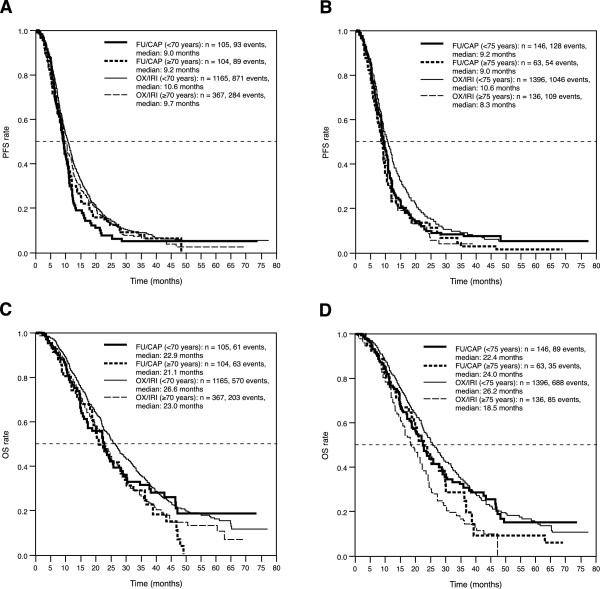
**Progression-free (PFS) and overall survival (OS) according to age and chemotherapy regimen. (A)** PFS according to age and chemotherapy regimen (<70 and ≥70 years); **(B)** PFS according to age and chemotherapy regimen (<75 and ≥75 years); **(C)** OS according to age and chemotherapy regimen (<70 and ≥70 years); **(D)** OS according to age and chemotherapy regimen (<75 and ≥75 years). FU/CAP: 5-fluorouracil-/capecitabine-based chemotherapy; OS: overall survival, OX/IRI: oxaliplatin-/irinotecan-based chemotherapy, PFS: progression-free survival.

Evaluation of PFS by chemotherapy regimen in patients <70 and <75 years of age showed PFS to be higher in those patients receiving an oxaliplatin- or irinotecan-based combination regimen compared with the respective groups receiving 5-FU/capecitabine (<70 years: 10.6 and 9.0 months, log-rank test p = 0.0065; <75 years: 10.6 and 9.2 months, log-rank test p = 0.028); however, no difference in PFS was observed between oxaliplatin- or irinotecan-based combinations and 5-FU/capecitabine regimens in both elderly age group comparisons (≥70 years: 9.7 and 9.2 months, log-rank test p = 0.52; ≥75 years: 8.3 and 9.0 months, log-rank test p =0.98; Figures 3A and B). The similar PFS observed with oxaliplatin/irinotecan regimens in the older age groups (≥70 and ≥75 years) compared with the younger age groups is likely to be the result of the selection of fewer but healthier patients better able to tolerate these regimens (for example, 66.0% of patients ≥75 years of age received oxaliplatin/irinotecan regimens versus 76.5% of patients ≥70 years of age).

Similarly, evaluation of OS by chemotherapy regimen and age found OS to be higher in patients <70 and <75 years of age receiving oxaliplatin/irinotecan compared with those receiving 5-FU/capecitabine (<70 vs. ≥70 years: 26.6 and 22.9 months, log-rank test p = 0.37; <75 vs. ≥75 years: 26.2 and 22.4 months, log-rank test p = 0.13), whereas there was no difference between oxaliplatin/irinotecan and 5-FU/capecitabine regimens was observed in patients ≥70 years of age (23.0 and 21.1 months, respectively; log-rank test p = 0.46; Figure 3C) and the outcome appeared to be reversed in patients ≥75 years of age (18.5 and 24.0 months, respectively; log-rank test p = 0.34; Figure 3D).

**Table 3:** **Overall investigator-assessed response rate to bevacizumab-based therapy according to patient age**

Response, n (%)	Age <70 years (n = 1297)	Age ≥70 years (n = 480)	Age <75 years (n = 1571)	Age ≥75 years (n = 206)
Complete response	104 (8)	34 (7)	139 (9)	17 (8)
Partial response	645 (50)	209 (44)	734 (47)	77 (37)
Stable disease	348 (27)	139 (29)	461 (29)	72 (35)
Progressive disease	96 (7)	45 (9)	115 (7)	17 (8)
Not evaluable	41 (3)	12 (3)	122 (8)	23 (11)

### Tolerability

In total, 270 potentially treatment-related adverse reactions were reported; of these 72 (27%) were considered to be serious (21% patients aged <70 years and 45% in those aged ≥70 years; 26% patients aged <75 years and 30% in those aged ≥75 years). The incidence of treatment-related adverse events was similar in patients aged <70, ≥70, <75 years and ≥75 years (Table 4).

**Table 4:** **Treatment-related adverse events (≥0.4%) according to patient age**

Adverse event, n (%)	Age <70 years (n = 1297)	ge ≥70 years (n = 480)	Age <75 years (n = 1571)	Age ≥75 years (n = 206)
Diarrhoea	36 (2.8)	13 (2.7)	45 (2.9)	4 (1.9)
Nausea	29 (2.2)	4 (0.8)	30 (1.9)	3 (1.5)
Hypertension	15 (1.2)	8 (1.7)	23 (1.5)	0
Bleeding	17 (1.3)	4 (0.8)	18 (1.1)	3 (1.5)
Leucopenia	7 (0.5)	1 (0.2)	8 (0.5)	0
Infection	6 (0.5)	2 (0.4)	8 (0.5)	0
Proteinuria	5 (0.4)	2 (0.4)	5 (0.3)	2 (1.0)
Phlebitis/thrombosis/embolism	11 (0.8)	6 (1.3)	15 (1.0)	2 (1.0)

The rate of premature withdrawal from the study was similar in both age group comparisons (<70 years 52% vs. ≥70 years 50%; <75 years 48% vs. ≥75 years 52%). More patients ≥75 years of age withdrew from the study because of a serious adverse event (10% vs. 6% of patients aged <75 years) but no difference was observed in those aged <70 vs. ≥70 years (6% vs. 7%). More patients ≥70 or ≥75 years of age withdrew because of treatment refusal (9% vs. 5%, and 10% vs. 6%, respectively), while more patients aged <70 and <75 years withdrew from the study because of planned surgery (7% vs. 4%, and 7% vs. 1%, respectively).

## Discussion

The ITT population in this observational study had a median PFS of 10.2 months, which is consistent with those reported in the phase III trials by Hurwitz *et al.* [6] and Saltz *et al.* [8] (10.6 and 9.4 months, respectively), and the Bevacizumab Expanded Access Trial (BEAT) study [14], the BRiTE study [12], the Avastin® Registry: Investigation of Effectiveness and Safety (ARIES) study [15], the Panitumumab Advanced Colorectal Cancer Evaluation (PACCE) study [16], and the HORIZON III study [17] (10.8, 9.9, 10.3, 10.5 and 10.3 months, respectively). Other observational cohort studies have reported median PFS values in the same range as our findings [18, 19].

Evaluation of PFS and OS by patient age found both to be significantly greater in patients aged <70 vs. ≥70 years and <75 vs. ≥75 years, despite the fact that median age at primary diagnosis of colorectal cancer in Germany is 69 years [20]. While the OS data are in agreement with findings from the BRiTE study, in which median OS was seen to decrease from 24.6 months in patients <65 years of age to 16.8 months in those >80 years [12], the decrease in PFS with increasing age was not observed in either the BRiTE study [21] or the bevacizumab pooled analysis [11]. Interestingly, in ARIES, a US observational cohort study, there were slight reductions in PFS (10.3 vs. 9.9 months) and OS (25.1 vs. 19.6 months) in mCRC patients ≥70 years compared with those <70 years of age receiving bevacizumab and chemotherapy in the first-line setting, but neither PFS (7.9 vs. 7.9 months) nor OS (18.7 vs. 17.2 months) differed in the second-line setting when comparing the two age categories [22, 23]. Furthermore, in the randomised AGITG MAX study, the improvement in PFS observed when bevacizumab was added to the existing chemotherapy regimen was similar in those patients ≥75 years of age compared with younger patients [24].

Overall, this analysis of elderly patients who participated in a large, community-based German observational cohort study shows that the use of bevacizumab plus chemotherapy is an effective first-line treatment option for patients with mCRC, independent of age. Nevertheless, as would be expected, median PFS and OS were significantly longer in patients aged <70 and <75 years than in the respective older patient groups, but there was no significant difference in the duration of bevacizumab therapy between the two age group comparisons. One possible explanation for this is that, despite both age groups being well balanced regards to most baseline characteristics, patients ≥70 or ≥75 years of age had a poorer ECOG performance status compared with the respective younger age group. This is likely to result in a more conservative, less intensive, approach to selecting the chemotherapy regimens for older patients (see below), as well as – for interpretation of the OS – less frequent use of all available drugs in subsequent treatments. The elderly group might also have a poorer general prognosis and certainly have a higher probability of non-tumour-related death.

The possibility that patients in the older age groups received less intensive therapy is supported when comparing the first-line chemotherapy regimens used. Fewer patients aged ≥75 years received bevacizumab plus doublet combinations at the start of treatment (60% vs. 86% of patients aged <75 years) while more patients aged ≥75 years received bevacizumab plus 5-FU or capecitabine alone (31% vs. 9% of patients aged <75 years). Evaluation of outcome according to chemotherapy regimen and age found that there were no differences in PFS and OS between age groups when treated with bevacizumab plus fluoropyrimidine monotherapy, but both PFS and OS were longer in patients aged <70 vs. ≥70 years and <75 vs. ≥75 years when treated with bevacizumab plus doublet combination chemotherapy. While the comparative efficacy in terms of PFS and OS of bevacizumab plus fluoropyrimidine monotherapy in younger and older patients appears to be in line with that reported in the ARIES study [21], the reason for the lack of increase in both PFS and OS with bevacizumab plus doublet chemotherapy in patients ≥75 years of age remains unclear. It is possible that these findings are the result of the lower numbers of patients in each subgroup, or that these particular patients had a worse prognosis.

Observational cohort studies allow the collection of data on the ‘real-world’ incidence and time to onset of treatment-related adverse events in general clinical practice, together with monitoring for the occurrence of any new safety signals. The incidence of treatment-related adverse events was similar in patients aged <70 vs. ≥70 years and those <75 vs. ≥75 years, as was the incidence of events considered to be serious. The rate of premature withdrawal from the study was similar in both age groups, but more patients the older age groups withdrew because of a serious adverse event or treatment refusal while more patients in the younger age groups withdrew because of planned surgery. The rate of adverse events of interest for bevacizumab was low in this study of German patients and was, overall, less than those reported in the BEAT [14] and BRiTE studies [21]; indeed, no increase in the incidence of arterial thromboembolosim in elderly patients was observed here in contrast to the increases reported in the pooled analysis of four randomised studies [11] and the BRiTE study [21]. It is possible that these differences could be the result of the way in which adverse event data were retrieved. Overall, no new safety signals were detected.

There are limitations that must be considered when interpreting findings from observational studies, with their inherent bias when selecting patients for specific treatment and maintenance strategies and when assessing progression and response. Nevertheless, minimal patient selection criteria were used and all patients scheduled to be treated with bevacizumab in the post-approval period were included in the study. Consequently, the population is more likely to be representative of the general oncology population in Germany (as is shown by the age distribution), and the study provides valuable information on the use of bevacizumab under the conditions and specifications of the German healthcare system.

This observational study only documented patients treated with bevacizumab; it might have been of interest to include a record of the reasons for which patients were excluded from bevacizumab therapy, but this was beyond the scope of the study.

## Conclusion

First-line bevacizumab-based treatment combinations were used successfully in German patients with mCRC, and also specifically in individuals ≥75 years of age. Findings from the current analysis suggest that for medically fit elderly patients chemotherapy doublets with bevacizumab may be regarded as a reasonable treatment option, although this should be interpreted with caution as the randomised, open-label FOCUS2 trial reported that the addition of oxaliplatin to fluoropyrimidine therapy versus no addition did not provide a significant improvement in PFS [10]. However, in line with findings from the AVEX trial [9], monotherapy using 5-FU or capecitabine in conjunction with bevacizumab was shown to result in promising PFS and OS data in elderly mCRC patients.
